# Rural Interfacility Emergency Department Transfers: Framework and Qualitative Analysis

**DOI:** 10.5811/westjem.2020.3.46059

**Published:** 2020-07-09

**Authors:** Candace D. McNaughton, Kemberlee Bonnet, David Schlundt, Nicholas M. Mohr, Suemin Chung, Peter J. Kaboli, Michael J. Ward

**Affiliations:** * Vanderbilt University Medical Center, Department of Emergency Medicine, Nashville, Tennessee; † Tenessee Valley Healthcare System, Department of Emergency Medicine, Nashville, Tennessee; ‡ Evaluation (CADRE) Iowa City VA Healthcare System, Center for Access & Delivery Research and Evaluation, Iowa City, Iowa; § University of Iowa Carver College of Medicine, Department of Emergency Medicine, Iowa City, Iowa; ¶ University of Iowa Carver College of Medicine, Department of Anesthesia, Iowa City, Iowa; || University of Iowa Carver College of Medicine, Department of Internal Medicine, Iowa City, Iowa

## Abstract

**Introduction:**

Interfacility transfers from rural emergency departments (EDs) are an important means of access to timely and specialized care.

**Methods:**

Our goal was to identify and explore facilitators and barriers in transfer processes and their implications for emergency rural care and access. Semi-structured interviews with ED staff at five rural and two urban Veterans Health Administration (VHA) hospitals were recorded, transcribed, coded, and analyzed using an iterative inductive-deductive approach to identify themes and construct a conceptual framework.

**Results:**

From 81 interviews with clinical and administrative staff between March–June 2018, four themes in the interfacility transfer process emerged: 1) patient factors; 2) system resources; and 3) processes and communication for transfers, which culminate in 4) the location decision. Current and anticipated resource limitations were highly influential in transfer processes, which were described as burdensome and diverting resources from clinical care for emergency patients. Location decision was highly influenced by complexity of the transfer process, while perceived quality at the receiving location or patient preferences were not reported in interviews as being primary drivers of location decision. Transfers were described as burdensome for patients and their families. Finally, patients with mental health conditions epitomized challenges of emergency transfers.

**Conclusion:**

Interfacility transfers from rural EDs are multifaceted, resource-driven processes that require complex coordination. Anticipated resource needs and the transfer process itself are important determinants in the location decision, while quality of care or patient preferences were not reported as key determinants by interviewees. These findings identify potential benefits from tracking transfer boarding as an operational measure, directed feedback regarding outcomes of transferred patients, and simplified transfer processes.

## INTRODUCTION

The emergency department (ED) is a central access point for healthcare in the United States. More than 60% of hospitalizations originate in the ED, which has become a default location for specialty consultations and diagnostic evaluations. This is particularly the case in rural areas, where hospitals have disproportionately closed in the past decade.^1^,^2^ As a result, interfacility transfers, in which a patient is transferred from an ED to another ED or hospital, are becoming a more common pathway to access care, even for time-sensitive emergencies.^3^–^6^

Rural ED visits to non-Veterans Health Administration (VHA) hospitals rose by more than 50% from 2006 to 2015, and a quarter of the ~4.2% patients who were transferred traveled more than 50 miles, most commonly for cardiovascular conditions.^7^,^8^ Rural patients at VHA EDs and urgent care clinics (UCC) are three times more likely to undergo interfacility transfers than their non-rural counterparts, and the most common reasons are for mental health conditions (34%), followed by cardiovascular conditions (12%; internal VHA data). Prolonged transfer times are common, and patients and their families often bear significant travel and economic burdens.^9^,^10^ This is particularly relevant in the VHA, where some rural healthcare facilities have limited clinic or specialty resources but maintain an ED or UCC. The 2018 VA MISSION Act included a new requirement to cover non-VHA urgent care access to care, further adding urgency to the need to better understand interfacility transfers.^11^ Therefore, to inform the design and implementation of a planned intervention to address interfacility transfers, we sought to understand the interfacility transfer process and identify and explore facilitators, barriers, and their implications for acute care access for rural veterans.

## METHODS

We conducted qualitative analysis of semi-structured interviews at multiple VHA facilities. We interviewed staff, clinicians, and administrators at seven VHA hospitals that accept and transfer patients from their EDs from March–June 2018 in accordance with COnsolidated criteria for REporting Qualitative (COREQ) research guidelines.^12^,^13^

### Recruitment and Data Collection

After piloting within the research team, semi-structured interviews (Supplement) were conducted by CDM and MJW, who are both emergency physicians and researchers with experience conducting interviews and qualitative analysis. Sites were chosen from 140 VHA ED/UCCs based on the proportion and number of ED/UCC visits that involved an interfacility transfer, as well as support from local leadership for conducting interviews, and geographic distribution. Staff were notified of the project by a local leader. We used purposeful and snowball sampling strategies to identify experienced stakeholders on both day and night shifts, including physicians, advanced practice providers, nurses, technicians, clerks, hospitalists, transfer coordinators, and clinical leadership.^14^ Interviews conducted at facilities were audiorecorded and transcribed, all identifiable information was removed, and field notes were reviewed for context and themes after each interview day. Recruitment ended when both agreed no additional information was being obtained. No repeat interviews were conducted. The Tennessee Valley Healthcare System internal review board determined these activities were quality improvement, and accordingly informed consent was obtained from each participant but not documented.

**Population Health Research Capsule**

What do we already know about this issue?

*Interfacility transfers from rural emergency departments are an important means of access to timely and specialized care*.

What was the research question?

*Identify and explore facilitators and barriers in transfer processes and their implications for emergency rural care and access*.

What was the major finding of the study?

*Patient factors, system resources, processes and communication all determine where patients are transferred*.

How does this improve population health?

*Interfacility transfers from rural EDs are complex, resource-driven processes. Transfer boarding should be tracked, and simplified transfer processes are needed*.

### Qualitative Analysis

We used an iterative, inductive-deductive approach to develop a conceptual framework for interfacility transfers at VHA facilities of different sizes in urban and rural locations.^15^ Deductively, we started with a framework developed in previous qualitative transfer work,^16^ combined with historical knowledge of ED processes. Inductively, we reviewed 10 interviews to refine categories and subcategories, and to develop higher order themes and relationships among themes. Four members of the team (CDM, MJW, KB, and DS) refined the coding framework until consensus was achieved. After a 10% random sample of transcribed interviews revealed no revisions to the coding framework, KB and SC recoded the preliminary set and remaining interviews.

## RESULTS

We conducted 81 interviews at two urban and five rural VHA hospitals, with 5–15 ED beds each (Table 1).

**Figure 1:**
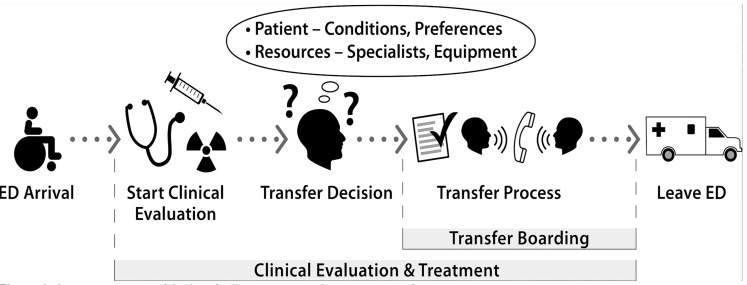
Core components of the interfacility emergency department transfer process.

**Figure 2:**
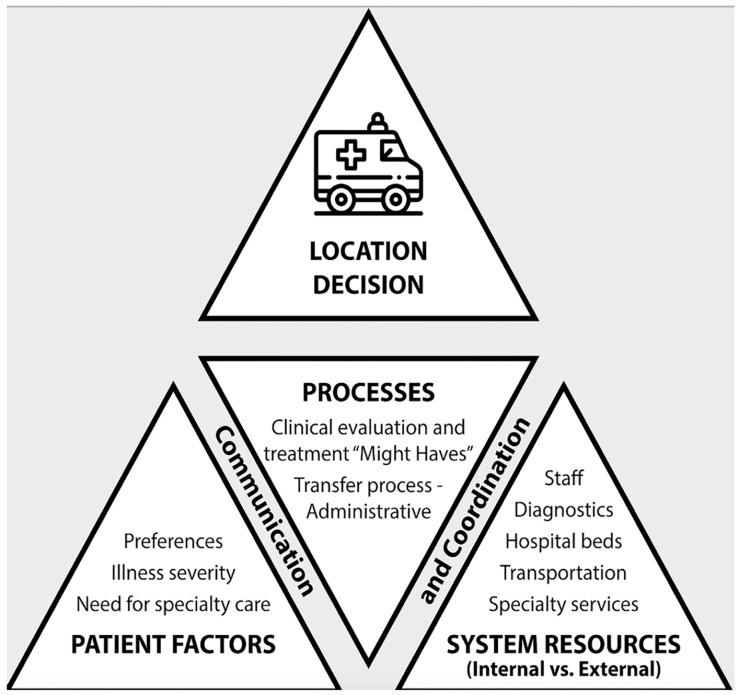
Interfacility emergency department (ED) transfer conceptual framework, with complex interplay among the central themes of 1) patient factors; 2) system resources; and 3) processes and communication among ED and non-ED clinicians, nurses, staff, which together culminate in 4) location decision, i.e., where, how, and when an ED patient is transferred.

Interviews were conducted among ED clinicians (N = 26), nurses (N = 24), and other staff such as clerks and respiratory therapists (N = 5); non-ED staff included administrators (N = 13), hospitalists (N = 11), and others (N = 4). No participants declined or dropped out; interviews were 10–45 minutes long. Interviews revealed core components of the interfacility ED transfer process, which are illustrated in Figure 1 and followed by a brief description. This process is made more complex by four themes that emerged from the interviews, which make up the conceptual framework (Figure 2) and have implications for access to care: 1) patient factors; 2) system resources; and 3) processes and communication for transfers, which culminate in 4) location decisions (ie, where, how, and when an ED patient is transferred).

The interfacility transfer process (Figure 1) includes history, physical exam, and potentially diagnostic testing. Once the need for transfer is identified (Figure 2), administrative steps are performed by multiple team members, including obtaining administrative approval (in some cases) and patient consent to transfer, finding an appropriate accepting facility, completing necessary forms and orders, arranging transportation, and conducting handoffs. The patient receives treatment until leaving the ED (ie, during transfer boarding). For each central theme in the conceptual framework, components and barriers are highlighted with representative quotes.

### Patient Factors

Patient *need for specialty care*, *illness severity*, and *patient/family preferences* were important considerations in the interfacility ED transfer process and location decision (top, Figure 2). This decision sometimes involved clinicians outside the ED, including hospitalist(s), mental health, surgery, and/or intensivist providers, as also described under the section *System Resources*. Diagnosis and comorbid conditions that required specialty care contributed to transfer and location decisions. When this assessment involved multiple clinicians, the process became complex.

“If I have a straightforward patient, like a neurosurgical patient, that’s relatively easy [because we automatically transfer them].…They get more complicated as the hospitalists get involved…[and w]hen a patient might go to surgery here…we need to make sure that they will allow them to go under anesthesia here, and anesthesia has their own criteria.” [Emergency physician 1, Facility A]

Yet, even in the setting of available specialty care, multiple clinicians and staff participated in the determination of whether a patient’s illness severity merited transfer.

“[Our hospital] tends to err on the side of sending the patient out if there is any indication that this patient might anything of a severe nature, or if they feel the patient *will develop* [emphasis added] any complications during their stay.” [Emergency physician 1, Facility A]

Where possible, patient and family preferences were considered in the transfer decision and location, particularly for long distances or if the patient had received prior care at another facility.

“By and large, none of [the ED patients] want that [to be transferred]. Because it’s a long trip, they won’t really be able to have any family visit them while they’re down there; it’s four hours down there. … [Transferring] would definitely not be the veteran’s choice.” [Emergency physician 1, Facility D]

### System Resources

Each region included urban and rural, VHA and non-VHA hospitals of varying sizes and distances from each other, and with different of *hospital bed capacity, specialty services, diagnostics, staff, and transportation* (bottom, Figure 2), which might be partially or completely unavailable depending on the hour, shift, or day of week or fluctuating staffing, ED patient arrivals, and other resource demands.

Fluctuating hospital bed capacity due to bed, bed type, or nursing availability was reported as one of the most important drivers of transfers. Access to specialty and diagnostic services varied by time of day and day of week; determination of need or anticipated need for specialty care could involve non-ED clinicians and staff (see also, Patient Factors above, and Clinical Processes, below*)*.

“[ED transfer frequency] depends on our bed availability in the ICU and on the floors. Last week we did a lot of transferring because we had no ICU beds…and the floor wasn’t taking any patients last week due to staffing issues.” [ED Nurse 1, Facility A]

“We only have one person each of every specialty. So for example, let’s say the [gastroenterology] GI doctor is out this week. … My hands are tied at that point. I can’t hang on to a patient with a hemoglobin of 6 [milligrams per deciliter] without the GI doctor here, whether it’s Monday morning or Friday evening.” [Hospitalist 1, Facility G]

“We have [CT] available 24/7, and basic imaging. But as far as MRI, we only have that on Monday, Tuesday, and Wednesdays. A lot of times on Thursday and Fridays we would send them to [the next closest VHA facility].” [Hospitalist 1, Facility G]

Patients were evaluated and treated by a complex team that could include ED and non-ED physicians, nurse practitioners or physician assistants, nurses, respiratory therapists, clerks, administrators, and others; they shared responsibilities when necessary to meet clinical demands, although this diverted resources from other ED patients.

“ If it’s a Saturday, it’s you [the emergency physician alone]. [I]t’s literally like I’m going down and I’m banging a door to Radiology and saying, ‘I need you to burn these images onto a disk. I need them in 10 minutes.’” [Emergency physician 2, Facility D]

“We’ll have the one-to-one sitter tied up [with a mental health patient while we wait up to six hours for transportation to arrive]. … When [there are only two nurses on shift overnight] the nursing supervisor will come and help us and sit there and do one-to-one for us.” [ED Nurse 1, Facility G]

Type and timing of transportation was arranged by the transferring facility and determined by the patient’s clinical severity, local requirements (eg, secure transportation for mental health patients), and availability of local resources such as ambulance services and staff. At facilities with a contract with a single ambulance service, staff reported that transfer boarding was longer when they used the contract service compared to when they received approval to use local emergency medical service (EMS) transportation. For time-sensitive transfers, the ability to use ground or aeromedical EMS varied.

“[T]he only thing we’re approved to call 911 [for EMS transportation] for is STEMI.” [Emergency physician 2, Facility A]

Additional requirements for mental health transfers (eg, special transportation such as secure transportation or police vehicle) and concerns for staff safety were described as contributing to prolonged transfer boarding times and as diverting already limited resources from other ED patients.

“The biggest part with mental health [transfers] is our transportation. It takes hours and hours and hours to usually get them out of here.” [ED nurse 2, Facility G]

“If they [a mental health patient] are agitated…they get more agitated [while waiting to transfer.]” [Hospitalist 1, Facility G]

### Processes, Communication and Coordination

Multifaceted interactions between system resources and patient factors occurred through *clinical and administrative processes* that occurred via complex *communication and coordination* within and across facilities (center, Figure 2). Clinical evaluation was an ongoing process and could prompt transfer, but *anticipated* resource need and clinical course were also described as important drivers in the transfer decision. Several interviewees described these as “might have” situations, in which a patient *might have* a condition requiring specialist consultation or *might have* clinical deterioration in the next 48 hours. Flexibility on the part of the ED team was required for clinical management and decisions about when, where, and how to transfer a patient.

“I’ve had a couple of occasions where [the admitting hospitalist has] come down and said, ‘I’m not as comfortable with [admitting the patient here] as I thought’.” [Emergency physician, Facility G]

Institutional steps for coordinating resources and making transfer arrangements varied by facility but were described as burdensome, complex, and primarily the responsibility of the treating ED clinician because other team members varied by facility, day of week, and time of day. A minimum of four forms and multiple phone conversations, typically by the ED clinician, were required prior to transfer. Interviewees reported that considerable clinician time was diverted from clinical duties to these administrative tasks. If a potential accepting facility declined the transfer, the process started over.

“After I notify the administrative officer, then I notify the patient and collect their informed consent, and get a signature. And then [I complete 4 forms:] an…interfacility transfer note and a non-VA medical or surgical consult…and [the transportation form] and my [ED clinical] note.” [Emergency physician 1, Facility A]

“We do a paper consent. … Like 50% of my job is transfer[ring ED patients].” [ED nurse practitioner, Facility C]

“A significant portion of our clinical day is actually spent transferring patients out [including] obtaining the consent, which on the computer sometime can be laborious and time consuming, but in addition to that having to speak to multiple facilities and multiple providers to see if they will accept our patients.” [Emergency physician 1, Facility A]

“I’m frustrated filling out redundant forms, forms that I know if it wasn’t some antiquated computer system, everything could populate over.” [Emergency physician, G]

*Clear communication and coordination* within and across facilities were vital for timely identification of patients who needed transfer and completion of the complex administrative transfer step. Staff reported that handoffs and multiple communication methods (phone, in-person, texts, etc.) were common, as were barriers and pitfalls. While transfer coordinators simplified and streamlined the process, most transfers occurred after daytime shifts; at multiple facilities, communication and coordination therefore defaulted back to the ED clinician at the same time of day that ED demand peaked and its role expanded to include communication and coordination of patient flow throughout hospital.

“[W]e [in the ED] serve as a buffer…for the system.” [Emergency physician, Facility E]

“[D]epending on the part of the day, it’s different people who facilitate the transfer. After [4 pm], it’s the [administrator on duty], that’s just one person. During daytime hours, it can include social work assisting with the transfer, and it can include the transfer coordinators assisting with the transfer. The transfer coordinator can say, “This guy’s been accepted. We need X amount of paperwork and then they can travel” … Then when that’s cleared and we have an accepting physician, provider handoff has to happen and nurse handoff has to happen. We also have to communicate with EMS… It can be a lot of red tape.” [ED nurse, Facility B]

### Location Decision

The final transfer location decision depended upon a complex interplay among patient factors, resources, and the clinical and administrative processes. Historical experiences (eg, whether a transfer was likely to be requested by local hospitalists and which facilities were likely to accept transfers) were described as playing an important role in how individual team members approached their tasks and therefore contributed to the final transfer location decision. Clinicians and administrative staff involved in the transfer process said they had to maintain a sense of what services were available and where to go to get access to needed resources. Community-wide lack of capacity, particularly for mental health facilities, was described as a barrier to finding an accepting facility.

“You have to know the capacities of [the other hospitals] and what they can safely accept and not accept.” [Emergency physician 3, Facility A]

“One time all the psych hospitals were full, including ours, and I had to sit on [a mental health patient who needed to be transferred] down here [in the ED].” [Emergency physician 1, Facility A]

Staff reported that facilities with transfer centers were preferred because the transfer request process was faster and the results more predictable.

“Each of the major tertiary facilities has a transfer center, which greatly aids us, … Sometimes people call…the smaller local hospital here, which has fairly good specialty coverage. I don’t tend to call there…because it is an onerous process.” [Emergency physician 3, Facility D]

Interviewees reported that it was often easier to transfer to non-VHA facilities regardless of facility resources or distance, because non-VHA facilities were more likely to have transfer centers and beds available.

“It’s easier to get [transfers] accepted at those [non-VHA] hospitals now that most of them have transfer centers.” [Emergency physician 4, Facility A]

In light of prolonged time between the decision to transfer and leaving the ED delayed treatment, staff said they workarounds to find accepting facilities based on their prior experiences.

“[A recent] patient was here [in the ED] for…almost two days [while we tried to find an accepting facility] … In the meantime he wasn’t receiving any care that he needs, while we were just holding him and giving him his maintenance routine meds. Our [emergency] physician…had spent most of his shift…on the phone back and forth with [multiple hospitals to find an accepting facility].” [Emergency physician 5, Facility A]

“[While transferring a patient] I just try to go with the flow. And if we hit a roadblock, I just sort of float around it and go to the next option. Very rarely do I get stiffed completely.” [Emergency physician 1, Facility A]

## DISCUSSION

This qualitative study examined drivers of and processes for transferring rural emergency patients to other facilities. We conducted 81 interviews at seven geographically distinct VHA facilities and identified four key components of interfacility ED transfers: 1) patient factors; 2) system resources; and 3) processes and communication for transfers, which culminate in 4) location decisions. According to information from interviews, transfer decisions were based on actual and anticipated resource needs and were strongly influenced by the transfer process itself, with the goal of timely transfer via the least complex process. Perceived quality or outcomes at the receiving location or patient preferences were not reported by interviewees as primary drivers of location decisions, perhaps in part because outcomes and quality of care for transferred patients were rarely, if ever, known.

Several staff reported that they kept manual track of outcomes for transferred patients by calling accepting facilities days or weeks later, but they would prefer a systematic method for post-transfer feedback as a means of continuing to improve patient care. Transfer process details varied but were frequently described as overly burdensome and diverting resources away from clinical care, including care for other ED patients. Transfers were also recognized as a burden for patients and their families. Finally, mental health transfers were perceived as having particularly prolonged transfer-boarding times.

Although interfacility ED transfers make up a minority of overall ED patient volume, they were perceived as using a disproportionate amount of clinical time and resources because of burdensome administrative processes and complex communications. Although anticipated need for resources (eg, potential need for specialized care in the next several days) was a common reason for transfer, there was no formal process for learning whether such transfers improve patient outcomes.

Transferring location was heavily influenced by process complexity; simpler processes were highly favored. Notably absent was a discussion of perceived quality or outcomes at receiving hospitals. Although not the focus of these interviews, prior work has found that transfer practices are based on relationships^17^ rather than patient outcomes and quality.^18^ Simplified transfer processes that address patient and family preferences while also providing objective feedback on patient outcomes^16^ are needed to create a transfer environment that minimizes disruption caused by transfers while maximizing patient outcomes.

Mental health transfers were described as particularly challenging. This is highly relevant for the VHA, where suicide prevention is among the top priorities^19^ and a common reason for seeking emergency care. Between 2012–2014, mental health conditions were the sixth most common reason for VHA ED visits (~2 million ED visits/year) and the most common reason for ED transfer, comprising 40.9% of all VHA ED transfers (internal VHA data). Interfacility ED transfers appeared to be an important strategy to access urgent and emergent mental health resources; therefore, simplified transfer processes and alternative means to access emergent mental health care (eg, telehealth^20^) should be carefully considered as alternatives to ED transfers.

Systematic assessment of transfer boarding may provide an opportunity to measure facility performance and assess strategies to mitigate these waits. Rural veterans and rural VHA healthcare sites are particularly reliant upon interfacility transfers to access emergency care because rurality contributes to disparities in quality, appropriateness, and efficiency of unscheduled mental health care.^21^ Our interviews highlight the tradeoff between use of interfacility ED transfers to obtain access to emergency care at the cost of transfer boarding, which was perceived as compromising quality patient care and staffing. This was particularly noted for patients with mental health conditions because they occupied more clinical and physical resources for longer periods and experienced greater delays starting definitive treatment, and it is borne out in research examining the impact of rising emergency care demands for mental health.^22^ Admission boarding is a well-known marker of ED and hospital performance^23^; although several interviewees at different facilities described mental health transfer boarding lasting hours and even days, transfer boarding is not to our knowledge a common operational metric.

## LIMITATIONS

While the ED-to-ED interfacility transfer process was broadly similar across facilities, and interviews were conducted until saturation was achieved based on review by multiple research team members, it is possible that transfer processes and their associated facilitators and barriers may differ at other VHA facilities. While we strove for diversity in geography and demographics, our findings may not be generalizable to all VHA facilities. Local context, including other non-VHA facilities, and local policies play an important role in the transfer process and its barriers and facilitators. Reasons for transfer may also differ for VHA compared to non-VHA ED facilities; thus, further work is needed to understand the degree to which these results apply to non-VHA settings. Finally, despite use of standardized qualitative methods, interviews may be influenced by social desirability bias, friendliness bias, acquiescence bias, or recall bias. Future work using quantitative methods, eg, tracking ED boarding time, should be compared to these findings.

## CONCLUSION

Interfacility transfers are multifaceted, time-consuming processes that require complex coordination of patient factors and system resources. The transfer process itself and anticipated needs play important roles, rather than quality of care or patient preferences. Mental health transfers epitomize these challenges. Future steps to improve emergency care for rural patients should consider reporting transfer boarding as an operational measure, providing transfer outcome feedback, simplifying transfer processes, and developing alternative strategies to obtain access to specialty care.

## Supplementary Information



## Figures and Tables

**Table 1:** Description of facilities and interviewees regarding the emergency department interfacility transfer process.

Facility	ED/UCC beds	URH classification	Accepts EMS	ED clinician*	ED nurse	ED staff	Administrative staff	Hospitalist	Other	Total
A	8	Rural	Yes	7	4	1	1	3	0	16
B	15	Urban	Yes	3	3	0	1	2	2	9
C	5	Rural	Yes	3	6	1	4	2	1	17
D	14	Rural	No	3	3	0	3	2	0	11
E	13	Urban	Yes	3	5	1	2	0	0	11
F**	5	Rural	No	3	2	1	2	0	1	9
G	6	Rural	No	2	3	1	0	2	0	8

* Clinicians included board-certified and non-board-certified physicians; nurse practitioners; and physician assistants.

** An urgent care clinic.

*ED*, emergency department; *UCC*, urgent care clinic; *URH*, urban/rural/highly rural classification; *EMS*, emergency medical services.
