# Supplementary material for: Rural Interfacility Emergency Department Transfers: Framework and Qualitative Analysis
Source: West J Emerg Med. 2020 Jul 9;21(4):858–65. doi: 10.5811/westjem.2020.3.46059 (PMC7390588; doi:10.5811/westjem.2020.3.46059)
Supplement: Supplementary file 1 [file wjem-21-858-s001.docx]

**APPENDIX: Semi-structured Interview Guide**

**Introductory Questions**

1. Please describe your general responsibilities in the ED. (Probe: primarily work days vs. nights, changed over time)
2. Please describe how often your facility accepts transfers and how often it transfers patients. Which occurs more often?
3. By your best estimate, how many inter-facility transfers you have been involved with in the past month?
4. How often does your ED receive/send transfers from/to other EDs? (Probe: VA vs. non-VA)

**Key Questions**

**The next set of questions involve the inter-facility transfer processes at your ED.**

1. *Transfer out of the ED*
2. Can you walk me through the steps of transferring a Veteran from your ED to another location/institution (Probe: How did you learn what to do, how do you remember what to do, which steps are you responsible for? How has this changed over time?)
3. What are some reasons that Veterans are transferred from your ED to another facility? (Probe: specialty care, bed capacity; specific conditions such as strokes, lack of resources [CT])
4. Which facilities (and what kind of facilities) are Veterans commonly transferred to from your ED? (Probe: VA vs. non-VA, ED vs. inpatient, why are these locations chosen)
5. Describe any situation(s) that you know of or may have been involved in, in which a Veteran was transferred from your ED but could have stayed at your facility (Probe: condition, local care capability, provider)

Supplemental

1. Please tell me about 1-2 situations in which the transfer of Veterans that went particularly well. Poorly? (Probe: specific facility, provide an example)
2. Compare and contrast processes for transfers vs. admissions to the same hospital (Probe: boarding, change of shift)
3. Where in the process of transferring a Veteran from your ED might you run into a delay or difficulty?
4. Receive from another ED
5. When a Veteran is transferred from another facility do your ED, can you walk me through the steps for how that is done? (Probe: How did you learn what to do, how do you remember what to do, which steps are you responsible for? How has this changed over time? Is it different during daytime/weekday?)
6. What are some reasons that Veterans are transferred to your facility? (Probe: specialty vs. bed capacity)
7. Where are Veterans commonly transferred from? (Probe: VA vs. non-VA, ED vs. inpatient, why these locations)
8. Describe any situations that you know of or were involved in, in which a Veteran was transferred to your facility but could have stayed (Probe: conditions such as stroke or heart attach, facility capability, provider reasons)

Supplemental

1. Please tell me about 1-2 situations in which the transfer of Veterans to your facility went particularly well. Poorly? (Probe: specific facility, provide an example)

**Next, I would like to discuss the Communication that occurs for a Veteran who is being transferred** *(do for both sending out and receiving)*

1. What information do you provide/get to/from the receiving/sending staff members? (Probe: which staff members, specific keywords/things to say, timing of communication)
2. Please describe how communication differs for transferring vs receiving.
3. Please describe any challenges or things that help in sharing information with staff members at another center.
4. Do you receive/give any feedback (positive or negative) from a receiving facility after Veteran has been transferred? (Probe: how is this provided, like receiving, differ by facility) Does your facility provide feedback to facilities that have transferred Veterans to you?
5. After a Veteran leaves the ED, particularly one who is older (>65 years old), would you like to know what happens to him/her? If so, what would you like to know?

**Closing Questions**

1. In what way might the goals of receiving/sending staff be different from your goals?
2. What kind of staff are involved in arranging a Veteran transfer? In your opinion and/or experience, which of these staff are most critical for a successful transfer and why?
3. What other challenges have you seen or know of in the process of transferring Veterans to and/or from your facility that we have not talked about?
4. What would you most like to see improved in the inter-facility transfer process?
5. What else can you tell us that may be able to help us?

“Thank you for speaking with me today. Please let me know if you have any questions about our conversation.”
